# Supplementary material for: Patterns of Intron Gain and Loss in Fungi
Source: PLoS Biol. 2004 Nov 30;2(12):e422. doi: 10.1371/journal.pbio.0020422 (PMC532390; doi:10.1371/journal.pbio.0020422)
Supplement: Table S1 — Also available at http://genes.mit.edu/NielsenEtAl/. (4.3 MB ZIP). [file pbio.0020422.st001.zip › NielsenEtAl/html/932.html]

AN7606.1.NCU06804.1.MG03143.1.FG04302.1


```
 CLUSTAL W (1.82) Multiple Sequence Alignments - Introns Inserted


Sequence 1: MG03143.1	578 aa
Sequence 2: FG04302.1	640 aa
Sequence 3: NCU06804.1	641 aa
Sequence 4: AN7606.1	873 aa
Alignment Length: 894 aa
Number Identitical Residues: 294 aa
Alignment Score (without introns) 15750


MG03143.1 	---------------~--------------------------------------------
NCU06804.1	---MATQSDNYYDIV1IVGAGPVGLMLSTCLSRWGYKIKHIDNRPEPTATGRADGIQPRS
FG04302.1 	MSQSQSDRSPYYDIV1IVGAGPVGLMLSTCLARWGYKIKHIDNRAEPTPTGRADGIQPRS
AN7606.1  	--MTTDSYPKKYDIV~IVGAGPVGILLSLCMSRWGYKVKHIDNRPVPTATGRADGIQPRS
          	      .     .     .:.. .   : . :  . . .  .. . .:.:. :.. .. :

MG03143.1 	------MGLKSGIMKHKPARVFEVAFWDPSTADASKGITRTGTWASCPSFIDARYPFTTL
NCU06804.1	LDLLRNMGLKSAIMAHKPARVYEVAFWDP--PKTGGGIVRTGTWPSCPEFIDARYPFTTL
FG04302.1 	LDLLRNMGLKSAIMAHKPARVYEVAFWDP--ASSGNGIARTGTWASCPDFIDARYPFTTL
AN7606.1  	TEILRNLGLKRKIMAYDPAKVYDVSFWDP--RPDGSGIMRTGNWPSCPRFIDTRYPFTTL
          	 .   .:***  ** :.**:*::*:****     . ** ***.*.*** ***:*******

MG03143.1 	LHQGFIERVFLSDLEENGVHVQRPWTITGFTSDEKKNPNYPVEVQLKHLDG-TASETIRA
NCU06804.1	LHQGLIERVFIADLEKNGTEIQRPWTITGFTSDEKADPEYPVTVQLAHVDDSSKTETVKA
FG04302.1 	LHQGLIERVFISDLEKNGAQVQRPWTIKSFTSDEKSNPDYPVTVELEHVDG-TAQETVHA
AN7606.1  	VHQGKIETVFLDEIKKAGTTVERPWTIIGFKNDG-LDATYPVQVQLKCLDT-NVVETVRA
          	:*** ** **: :::: *. ::***** .*..*   :. *** *:*  :*  .  **::*

MG03143.1 	KYLFGGEGARSFIREQLGIGIKHKDPISHVWGVMDGVVQTDFPDIK0MKCTIHSRHGSIM
NCU06804.1	KYLFGGEGARSFIRDQLKIGITHKDPISYVWGVMDGVVKTDFPDMK~MKCTIHSEHGSIM
FG04302.1 	KYLFGGEGARSFIRNELGIAVHHKDPIAHVWGVMDGVVKTDFPDIK0MKCTIHSEHGSIM
AN7606.1  	KYLFSGEGARSFVREQLGIQIRHKDPISYVWGVMDGVVRTDFPDIQ0TKCTIHSDAGSIM
          	****.*******:*::* * : *****::*********:*****::  ******  ****

MG03143.1 	VIPRENNLVRLYIQIASSTDPDFNPRKTATEQEVQASAKKILQPYSIEWERVEWYSVYPI
NCU06804.1	VIPREANMVRLYIQIASSTDPDFHPRKTATEAEVQAAAKRILAPYSIEWERVEWYSVYPI
FG04302.1 	VIPREDNMVRLYIQIASSTDPDWNPRRTATEEQVQESAKKILSPYSIEWERVEWYSVYPI
AN7606.1  	VIPREDDMVRLYVQIASSSDPDFNPRKTATAEEVQNVAKKILKPYYIEWDRVEWYSVYPI
          	***** ::****:*****:***::**:***  :**  **:** ** ***:**********

MG03143.1 	GQGIADRYTLDHRVFLGGDSCHTHS0PKAGQGMNTAFLDGQNLAWKIHAVESGFAKRSLL
NCU06804.1	GQGISDKYTLDHRVFLGGDACHTHS~PKAGQGMNTAFLDALNLAWKIHAVEGGFADRHIL
FG04302.1 	GQGISEKYTLDQRVFLGGDACHTHS0PKAGQGMNTAFLDAQNLAWKIHAVESGFAKREIL
AN7606.1  	GQGISEKYTLDERVFMGGDACHTHS0PKAGQGMNTAFHDALNMAWKLHAVESGLAQRSIL
          	****:::****.***:***:***** *********** *. *:***:****.*:*.* :*

MG03143.1 	ETYESERKAVAETLLDFDNKYAKLFSTRPPDASEVKAAS----EDHGNQG-GHDNDFVRT
NCU06804.1	ETYEPERKDVAETLLAFDNKYAKLFSQRPDAASTAQATASGTHANPDAQQSAEENEFIKT
FG04302.1 	QTYETERKHVAESLLDFDNRYAKLFSQRPPAAAEVQAAS----ANHSKNGDAQENEFINT
AN7606.1  	STYETERKNIAETLLDFDNKYAALFSKRRPNAGEVGEAA-----TAETGRSAEEDPFVKT
          	.***.*** :**:** ***:** *** *   *. .  ::           ...:: *:.*

MG03143.1 	FKESCEFTSGYGVAYQPNQLNWSPSHP---AQSSTINPEG--NKLRPGRLFINADVTRVA
NCU06804.1	FKEACEFTSGYGVFYKPNALNYSPAHHPAGTSPTILSPEDQTTKLVPGRLFINSTVTRVV
FG04302.1 	FKESCEFTSGYGVYYKPNVFNWSPEHT---AQSPLIHPQG--TKLRTGRIFINSNVTRVV
AN7606.1  	FKDSCEFTSGYGVAYLPNIFNWDPSHP---AKSPLFDVPG--INLVTGKAFTPSTVTRLA
          	**::********* * ** :*:.* *    :... :   .   :* .*: *  : ***:.

MG03143.1 	DANVVHLEQEVPLNGSFRIFVFAG--KPSVTKQALSDFARNLRLKGSFFSSYLRSDIDKV
NCU06804.1	DANVVHLEQEIPLNGSFRLFIFAGSLTSPAQKSALESLAAGLQKPDSFFSRFQRPDIDTV
FG04302.1 	DANVVHLEQEVPLNGAFRLFVFAG--VPSKTRQALKDLASHLGSQRSFYAQYQRADLDKV
AN7606.1  	DSNFVHLEQEIPANGAFRIFIFAG--RQSRSKKAIADFAANLEKERSFLSAYRRSDIGEI
          	*:*.******:* **:**:*:***    .  :.*: .:*  *    ** : : *.*:. :

MG03143.1 	SYHERHNPHSKFFTFCTTFAAPRSSIEISRDVPDILARYSDHIYADDRIHREGRGAGPAM
NCU06804.1	SHHERHNPHSKFFTLATVFAAKRAEVEISRDLPALLARYRDNVYADDRWDR--RVPDAKA
FG04302.1 	SYHEKHLPHSRFFTLSTIFAARRPDIEISRDVPGVLARYRDHVYADDRSD--ARFAQAKA
AN7606.1  	SFFERHNPHSKLFTLCLIYAEKKNDIDMD-SIPQILRDYRYHIYSDDIPDV--RVPNATY
          	*..*:* ***::**:.  :*  : .:::. .:* :*  *  ::*:**  .   * . .  

MG03143.1 	SAHKKMGLDEERGGVVVVRPDGYVGVATKLVEGSGTVDALNQYFAAFCTQRTGAATAQL~
NCU06804.1	AAHAKMGFDEDKGGVVVVRPDGYVGFVCRLEEGGKTVEALEKYFGAFTTKQ--------~
FG04302.1 	VAHAKMGLDEELGGVVVVRPDGYVGVVVSLVEGSGTVDALNEYFSTFCTKKLGTTMAQL~
AN7606.1  	AAHEKLGFDPEKGGVVVTRPDSHIACTVQLAEGSGTVDALNAYFGSFSTKPLGQEQASP1
          	 ** *:*:* : *****.***.::. .  * **. **:**: **.:* *:  .   :.  

MG03143.1 	----------------~-----------------------------------~-------
NCU06804.1	----------------~-----------------------------------~-------
FG04302.1 	----------------~-----------------------------------~-------
AN7606.1  	YSIKCFPSFSRQSSQD1LRPQDTEEAPYYYTFKVQCTSCRETHPNWVSFNRF0STQEQHE
          	 : .. .: : .::..   ...:..:.   : . ..::. .: ..  : .   ::... .

MG03143.1 	------------------~-----------------------------------------
NCU06804.1	------------------~-----------------------------------------
FG04302.1 	------------------~-----------------------------------------
AN7606.1  	IPGSRGEANFVWKCKLCQ0RTHSASVLAGPNAYAAGDDKRKGQKIIDLECRGLEFTEFKP
          	 ..: ..:.   ... ..  : :::  :...: ::.... ....  . .. . . :. ..

MG03143.1 	-~-----------------------------------------------~----------
NCU06804.1	-~-----------------------------------------------~----------
FG04302.1 	-~-----------------------------------------------~----------
AN7606.1  	D0GEWEAVGVESSTPFKGIDLSEGEWYDYDEKAGDEVAIKEIKWELGRA2VGTEVIVRLK
          	. .. .: . .:::. .. . :...  . ...:... : .. . . . :  .:.     .

MG03143.1 	------------------------------------------~-----------------
NCU06804.1	------------------------------------------~-----------------
FG04302.1 	------------------------------------------~-----------------
AN7606.1  	WGQTEYKGRLESIDSYMNVLLRDTEEFIDGKPTGTLGLVLIR2CNNILWMGSAENVEMTD
          	 ..:. ..  .: .:  .    .:..  ....:.: .      ...    .::.. . :.

MG03143.1 	----
NCU06804.1	----
FG04302.1 	----
AN7606.1  	LGLR
          	 .
```
